# Effects of pecto-intercostal fascial block combined with rectus sheath block for postoperative pain management after cardiac surgery: a randomized controlled trial

**DOI:** 10.1186/s12871-023-02044-w

**Published:** 2023-03-23

**Authors:** Lu Wang, Luyang Jiang, Bailin Jiang, Ling Xin, Miao He, Wei Yang, Zhou Zhao, Yi Feng

**Affiliations:** 1 Department of Anesthesiology, Peking University People’s Hospital, 11 Xizhimen South Street, Beijing, 100044 China; 2 Department of Cardiac Surgery, Peking University People’s Hospital, Beijing, China

**Keywords:** Nerve block, Cardiac surgical procedures, Opioid, Analgesia

## Abstract

**Background:**

Pecto-intercostal fascial block (PIFB) provides analgesia for cardiac median sternotomy, but many patients complain of severe drainage pain that cannot be covered by PIFB. Rectus sheath block (RSB) has been attempted to solve this problem, but whether PIFB combined with RSB can achieve better analgesia is uncertain.

**Methods:**

This was a single-center randomized controlled trial at Peking University People’s Hospital from September 22, 2022 to December 21, 2022. Patients undergoing elective cardiac surgery with a median sternotomy were randomized at a 1:1 ratio to receive either bilateral PIFB and RSB (PIFB + RSB group) or PIFB (PIFB group). The primary outcome was intravenous opioid consumption within 24 h after surgery. Secondary outcomes included opioid consumption within 48 h, postoperative pain scores, time to extubation, and length of stay in the hospital. Interleukin (IL)-6, IL-10, and tumor necrosis factor (TNF)-α before and the first 24 h after surgery were measured.

**Results:**

A total of 54 patients were analyzed (27 in each group). Intravenous opioid consumption within 24 h after surgery was 2.33 ± 1.77 mg in the PIFB + RSB group vs 3.81 ± 2.24 mg in the PIFB group (*p* = 0.010). Opioid consumption within 48 h after surgery was also reduced in the PIFB + RSB group (4.71 ± 2.71 mg vs 7.25 ± 3.76 mg, *p* = 0.006). There was no significant difference in pain scores, time to extubation, length of stay in hospital, or the levels of IL-6, IL-10 and TNF-α between the two groups.

**Conclusions:**

The combination of PIFB and RSB reduced postoperative intravenous opioid consumption until 48 h after cardiac surgery.

**Trial registration:**

This trial is registered at the Chinese Clinical Trial Registry (www.chictr.org.cn, ChiCTR2200062017) on 19/07/2022.

## Background

Acute postoperative pain is severe in cardiac patients undergoing sternotomy, and pain intensity is more severe than expected [1]. Poorly controlled pain after surgery can lead to myocardial ischemia and pulmonary infections [2]. A perioperative multimodal opioid-sparing pain management plan is recommended to accelerate recovery [3]. Novel fascial regional techniques such as pecto-intercostal fascial block (PIFB) have been applied in cardiac surgery and have achieved satisfying analgesia without the consideration of heparinization [4, 5]. However, postoperative pain in cardiac surgery is a multidimensional phenomenon that involves incision, sternal retraction, musculoskeletal trauma and drainage catheter insertion sites. Many patients referred tube insertion as the most painful site after coronary artery bypass surgery [6]. Rectus sheath block (RSB) can offer somatic analgesia for midline incisions [7], and it has been verified to manage subxiphoid drainage pain effectively and safely for patients undergoing cardiac surgery [8].

We conducted a single-center randomized controlled trial to explore the hypothesis that PIFB combined with RSB, covering more surgical area, could reduce opioid consumption and achieve better analgesia after sternotomy.

## Methods

This trial was conducted at the Peking University People’s Hospital from September 22, 2022, to December 21, 2022. The study was approved by the Ethical Review Committee of Peking University People’s Hospital (#2022PHB179-001). Then, it was registered in the Chinese Clinical Trial Registry (ChiCTR2200062017) on 19/07/2022.

### Participants

Inclusion criteria included 1) elective cardiac surgery with a median sternotomy; 2) American Society of Anesthesiologists (ASA) II ~ III; and 3) adult patients (18 to 75 years of age). Exclusion criteria included 1) known allergy to ropivacaine; 2) platelet count < 100*10^9/L; 3) infection at the puncture site; 4) a history of opioid abuse; and 5) cognitive dysfunction and communication difficulties. Written informed consent was obtained from all participants.

### Randomization and blinding

Eligible patients were randomized at a 1:1 ratio to be allocated to the PIFB + RSB group (20 ml 0.3% ropivacaine plus 2.5 mg dexamethasone on each side for PIFB and 15 ml 0.3% ropivacaine plus 2.5 mg dexamethasone on each side for RSB) and PIFB group (20 ml 0.3% ropivacaine plus 2.5 mg dexamethasone on each side for PIFB and 15 ml normal saline on each side for RSB) after induction of general anesthesia before incision. A randomization sequence was generated using a personal computer with a block size of 6 before recruitment by a biostatistician. Concealment was conducted using opaque, sealed envelopes. The envelopes were delivered to another researcher, and he prepared ropivacaine or normal saline. All nerve block procedures were performed by an appointed researcher, and he did not learn about what the fluid was. Anesthesiologists, patients and follow-up nurses were blinded to the group allocation. Statistical analysis was performed by another researcher who was blinded to allocation.

### Anesthesia

Anesthesia was induced with 0.02–0.04 mg/kg midazolam, 0.2–0.4 mg/kg etomidate, 1–1.5 μg/kg sufentanil and 0.2–0.3 mg/kg cisatracurium. The bispectral index was maintained at 40–55 with propofol, sevoflurane, dexmedetomidine and cisatracurium. Bolus sufentanil (0.3–0.5 μg/kg) and vasoactive drugs were given by supervising anesthesiologists according to hemodynamic changes. After surgery, patients were transferred to the intensive care unit (ICU) for extubation and further medical care.

### Ultrasound-guided PIFB procedure

**Fig. 1:**
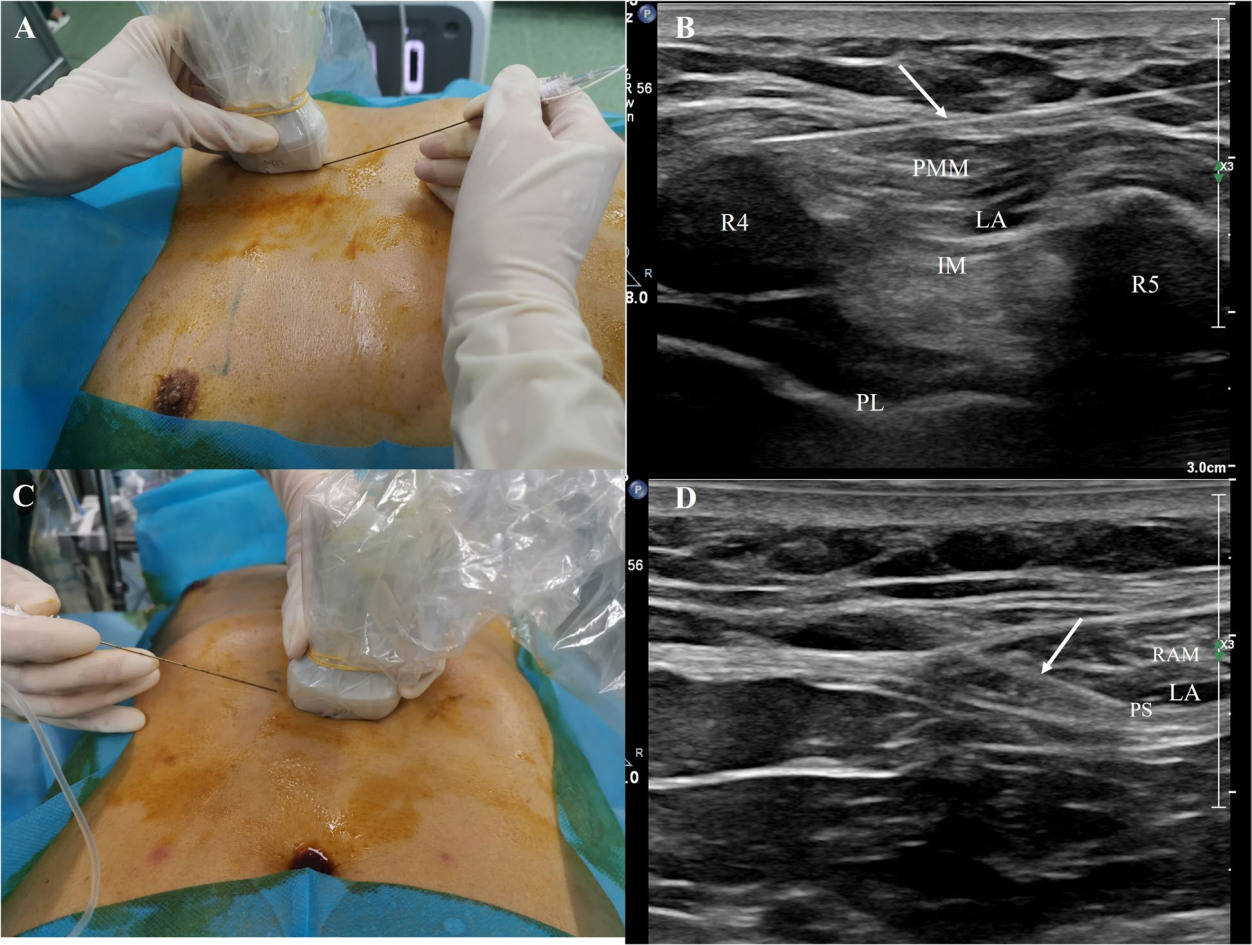
Procedures of PIFB and RSB. **A** Patient positioning, transducer and needle orientation during PIFB. **B** Anatomical location of PIFB on ultrasound and dissemination of local anesthetics. **C** Patient positioning, transducer and needle orientation during RSB. **D** Anatomical location of RSB on ultrasound and dissemination of local anesthetics. Abbreviations: IM-intercostal muscle, LA-local anesthetics, PIFB-pecto-intercostal fascial block, PL-pleura, PMM-pectoralis major muscle, PS- posterior sheath of RAM, R4-fourth rib, R5-fifth rib, RSB-rectus sheath block, RAM- rectus abdominis muscle. Needle was showed as the white arrow

Bilateral PIFB was conducted in the supine position under ultrasound guidance after anesthesia induction. A high-frequency linear ultrasound probe (EPIQ7C, PHILIPS, Holland) was placed 2–3 cm lateral to the edge of the sternum at the fourth intercostal space to identify anatomic landmarks (Fig. 1). A 21-gauge, 100 mm needle (SonoPlex STIM, PAJUNK, Germany) was inserted into the pecto-interfacial plane between the pectoralis major muscle and intercostal muscle using an in-plane technique. After verifying needle placement (visualizing the muscles separation upon injection of 2 ml saline), 20 ml 0.3% ropivacaine containing 2.5 mg dexamethasone was delivered to each side.

### Ultrasound-guided RSB procedure

Bilateral RSB was conducted after the PIFB was performed with the same position and probe. The probe was placed 2–3 cm next to the xiphoid in the epigastric region (Fig. 1). The needle was inserted into the plane between the rectus abdominal muscle and its posterior sheath using an in-plane technique. After verifying needle placement (visualizing the muscle separation upon injection of 2 ml saline), 15 ml 0.3% ropivacaine containing 2.5 mg dexamethasone was delivered to each side. Patients in the PIFB group received 15 ml normal saline.

### Postoperative analgesia

All patients received patient-controlled intravenous analgesia (PCIA) with a standard regimen of hydromorphone (no basal infusion, 0.2 mg bolus and 10-min lockout intervals). Patients were educated on how to evaluate pain intensity and use PCIA properly by professional staff the day before surgery. Postoperative pain was assessed using a 10-point numeric rating scale (NRS) at rest and cough at 12, 24 and 48 h after surgery. An oral polypill consisting of oxycodone (5 mg) and acetaminophen (325 mg) was given as rescue analgesia for moderate to severe pain (pain score of 4 or greater at any time within 48 h). Intravenous tropisetron (5 mg) was used to treat postoperative nausea and vomiting (PONV).

### Outcomes and biochemical parameters

The primary outcome was intravenous opioid consumption at 24 h after surgery. Secondary outcomes included intravenous opioid consumption within 48 h, intraoperative opioid consumption, pain score at rest and upon coughing at 12, 24 and 48 h, moderate-to-severe pain, pain at the drainage within 48 h, creatine kinase-MB and cardiac troponin I at 24 h, time to extubation, time to drainage removal, time to catheter removal, ability to ambulate after surgery, length of stay (LOS) in the ICU and hospital, mortality within 30 days and incidence of chronic pain at three months after surgery. Opioid-related adverse events included PONV, urinary retention, dizziness and pruritus within 48 h. Interleukin (IL)-6, IL-10 and tumor necrosis factor (TNF)-α were measured before the induction of anesthesia and 0 and 24 h after surgery. Opioid consumption within 48 h after surgery only included hydromorphone in the PCIA. Whole blood was immediately centrifuged at 3000 rpm for 10 min to separate the plasma. Then, it was frozen at -80 °C for subsequent analysis.

### Statistical analysis

Sample size estimation was based on our pilot trial: 1) opioid consumption at 24 h after surgery was 2.1 ± 1.4 mg in the PIFB + RSB group and 3.4 ± 1.9 mg in the PIFB group (*n* = 7 in each group); 2) α at 0.05 and β at 0.20; and 3) dropout rate of 10%. The calculation yielded 60 subjects (30 in each group). Continuous variables other than pain score were analyzed using Student’s t test. The pain score was analyzed using analysis of variance for repeated measures with Geisser-Greenhouse correction and Sidak’s test for comparisons at each time point. Categorical variables were analyzed using Fisher’s exact test. Statistical significance was defined as *p* < 0.05 (2-sided). All statistical analyses were performed using GraphPad Prism version 9.0.

## Results

**Fig. 2:**
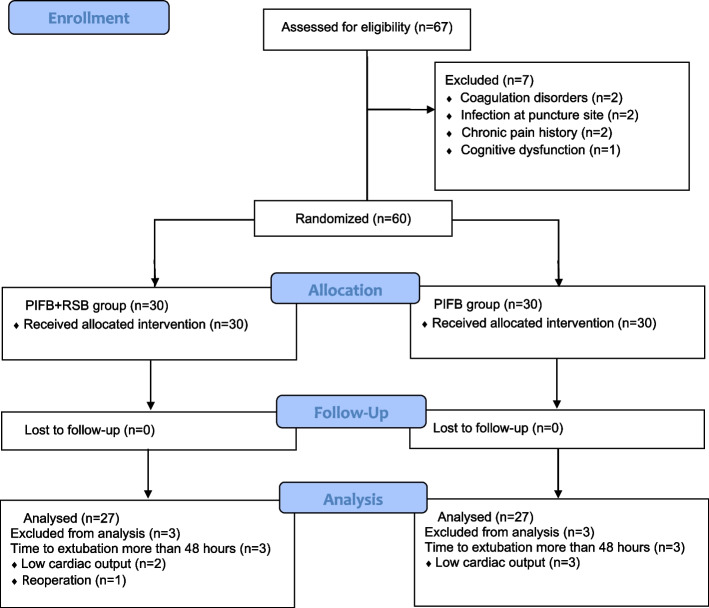
Patient flow through the trial

A total of 67 patients were screened, 60 (mean age: 62.81 ± 8.59 years; 36 men) were randomized, and 54 were analyzed (27 in each group). Six patients (5 patients with low cardiac output, 1 patient with reoperation) were excluded because of late extubation, as they did not use opioids within 48 h after surgery (Fig. 2). The demographic and baseline characteristics of the participants are shown in Table 1.

**Table 1:** Demographic and baseline characteristics

	PIFB + RSB group (*n* = 27)	PIFB group (*n* = 27)
Age, mean ± SD, year	63.07 ± 7.11	62.96 ± 8.59
Male, n (%)	14 (51.9)	16 (59.3)
BMI, mean ± SD, kg/m^2^	24.88 ± 3.26	24.99 ± 3.90
Comorbidities, n (%)		
Hypertension	18 (66.7)	18 (66.7)
Diabetes mellitus	11 (40.7)	12 (44.4)
Cerebral infarction	3 (11.1)	5 (18.5)
Asthma	2 (7.4)	1 (3.7)
ASA, n (%)		
II	2 (7.4)	2 (7.4)
III	25 (92.6)	25 (92.6)
NYHA, n (%)		
II	2 (7.4)	2 (7.4)
III	25 (92.6)	25 (92.6)
EF before surgery, mean ± SD, %	62.10 ± 10.9	63.08 ± 10.13
Operation with CPB, n (%)	15 (55.6)	14 (51.9)
Duration of CPB, mean ± SD, min	189.08 ± 38.84	172.51 ± 33.29
Surgery type, n (%)		
Single CABG	18 (66.7)	17 (63.0)
Single valve surgery	5 (18.5)	7 (25.9)
Combined procedures	4 (14.8)	3 (11.1)
Surgical time, mean ± SD, min	320.48 ± 61.29	291.52 ± 67.47
Anesthetic time, mean ± SD, min	415.04 ± 64.06	382.59 ± 72.19
EuroSCORE II, median (IQR)	1.61 (0.99 to 2.95)	1.60 (1.00 to 2.92)

*ASA* American Society of Anesthesiologists, *BMI* body mass index, *CABG* Cardiac artery bypass graft, *CPB* Cardiopulmonary bypass, *EF* Ejection fraction, *EuroSCORE II* European System for Cardiac Operative Risk Evaluation II, *IQR* Interquartile range, *NYHA* New York Heart Association, *PIFB* Pecto-intercostal fascial block, *RSB* Rectus sheath block, *SD* Standard deviation

**Fig. 3:**
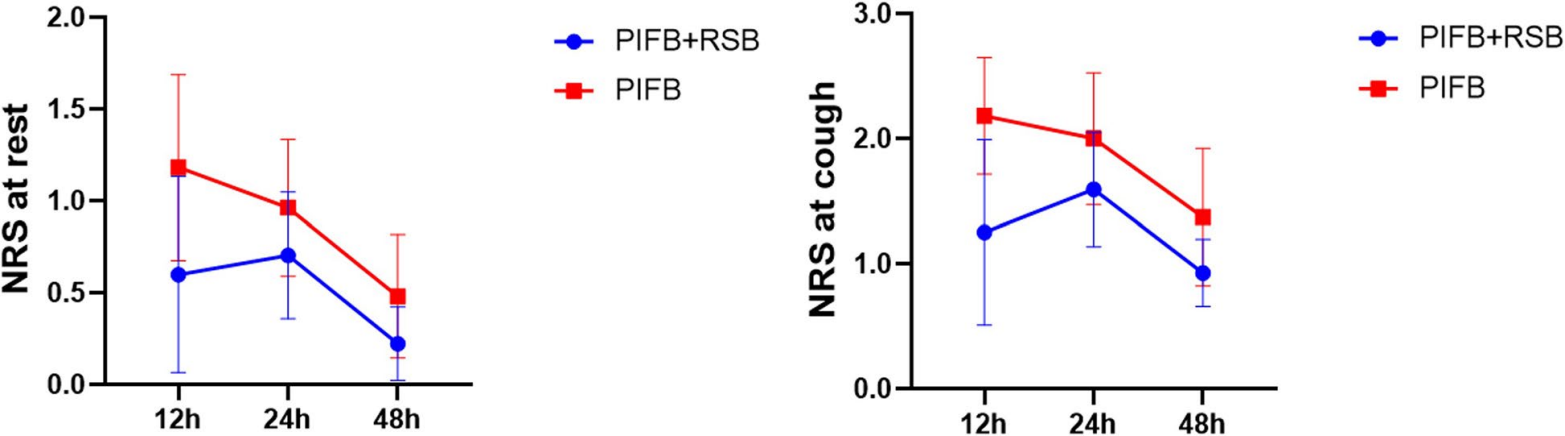
Postoperative pain score. **A** at rest. **B** at cough. Data were analyzed using 2-way ANOVA, followed by Sidak’s multiple comparisons for each time point. Data are shown as the mean ± 95% confidence interval. *p < 0.05. Abbreviations: NRS-numeric rating scale, PIFB-pecto-intercostal fascial block, RSB-rectus sheath block

Intravenous opioid consumption at 24 h after surgery was 2.33 ± 1.77 mg in the PIFB + RSB group vs 3.81 ± 2.24 mg in the PIFB group (*p* = 0.010). Intravenous opioid consumption at 48 h after surgery was 4.71 ± 2.71 mg in the PIFB + RSB group vs 7.25 ± 3.76 mg in the PIFB group (*p* = 0.006). The pain score did not differ between the two groups at rest (*p* = 0.287, 0.653, 0.449) or cough (*p* = 0.097, 0.551, 0.371) at 12, 24 and 48 h (Fig. 3). Intraoperative sufentanil consumption was similar (166.30 ± 40.54 vs 163.70 ± 41.06 μg, *p* = 0.816). There was no difference in adverse events with opioids (29.6% vs 33.3%, *p* = 0.770). The incidence of pain at the chest tube within 48 h was 14.8% in the PIFB + RSB group and 29.6% in the PIFB group (*p* = 0.190). The incidence of chronic pain at three months was 18.5% and 25.9% in the PIFB + RSB group and PIFB group, respectively (*p* = 0.513). (Table 2) The two groups did not differ in time to extubation, time to drainage removal, time to catheter removal, ability to ambulate, or LOS in the ICU or hospital. All participants in the two groups survived at 30 days after surgery. No adverse events were found related to regional block procedures in the trial. There were no significant differences in the levels of IL-6, IL-10, and TNF-α between the two groups at baseline or 0 and 24 h after surgery. (Table 3).

**Table 2:** Primary and secondary outcomes

	PIFB + RSB group (*n* = 27)	PIFB group (*n* = 27)	p
Opioid consumption within 24hs, Mean ± SD, mg	2.33 ± 1.77	3.81 ± 2.24	0.010*
Opioid consumption within 48hs, Mean ± SD, mg	4.71 ± 2.71	7.25 ± 3.76	0.006*
Intraoperative sufentanil consumption, Mean ± SD, μg	166.30 ± 40.54	163.70 ± 41.06	0.816
Moderate to severe pain, n (%)	5 (18.5)	10 (37.0)	0.129
Pain at the chest tube within 48 h, n (%)	4 (14.8)	8 (29.6)	0.190
Adverse events with opioid, n (%)	8 (29.6)	9 (33.3)	0.770
nausea and vomiting	6 (22.2)	6 (22.2)	1.000
pruritus	0 (0)	1 (3.7)	-
urinary retention	1 (3.7)	0 (0)	-
dizziness	1 (3.7)	2 (7.4)	1.000
Creatine kinase-MB at 24 h, median (IQR), ng/ml	6.50 (3.00–19.40)	7.10 (2.40–20.90)	0.842
Cardiac troponin I at 24 h, median (IQR), pg/ml	1074.10 (240.30–4076.20)	878.80 (347.40–5013.70)	0.829
Time to extubation, Mean ± SD, hour	9.12 ± 5.10	9.90 ± 5.06	0.578
Length of stay in ICU, Mean ± SD, hour	53.51 ± 40.48	52.76 ± 43.87	0.949
Time to drainage removal, Mean ± SD, hour	78.14 ± 23.31	77.21 ± 20.03	0.876
Time to catheter removal, Mean ± SD, hour	91.18 ± 35.02	95.31 ± 37.06	0.675
Able to ambulate after surgery, Mean ± SD, hour	92.42 ± 37.07	92.33 ± 38.63	0.993
Length of hospital stay, Mean ± SD, day	12.22 ± 6.62	12.56 ± 6.28	0.850
Mortality within 30 days after surgery, n (%)	0 (0)	0 (0)	-
Chronic pain at three months, n (%)	5 (18.5)	7 (25.9)	0.513

*ICU* Intensive care unit, *IQR* Interquartile range, *PIFB* Pecto-intercostal fascial block, *RSB* Rectus sheath block, *SD* Standard deviation

**Table 3:** Measures of blood markers

	PIFB + RSB group (*n* = 27)	PIFB group (*n* = 27)	p
IL-6, Mean ± SD (pg/ml)			
baseline	2.34 ± 1.41	2.51 ± 1.54	0.965
0 h after surgery	62.09 ± 62.81	54.72 ± 44.40	0.947
24 h after surgery	76.05 ± 69.13	130.48 ± 159.99	0.306
IL-10, Mean ± SD (pg/ml)			
baseline	3.01 ± 0.96	2.92 ± 0.96	0.979
0 h after surgery	83.01 ± 117.98	84.10 ± 122.99	0.999
24 h after surgery	3.92 ± 2.64	3.42 ± 1.25	0.769
TNF-α, Mean ± SD (pg/ml)			
baseline	2.97 ± 1.01	3.12 ± 1.49	0.966
0 h after surgery	2.95 ± 1.46	2.58 ± 1.49	0.749
24 h after surgery	3.38 ± 1.40	2.99 ± 1.80	0.759

*IL* Interleukin, *PIFB* Pecto-intercostal fascial block, *RSB* Rectus sheath block, *SD* Standard deviation, *TNF* Tumor necrosis factor

## Discussion

This trial demonstrated that PIFB combined with RSB can reduce intravenous opioid consumption until 48 h after cardiac surgery and did not reduce perioperative systemic inflammation.

Intravenous opioid consumption within 24 h after surgery was significantly decreased in the PIFB + RSB group (2.33 ± 1.77 mg vs 3.81 ± 2.24 mg, *p* = 0.010). Opioid consumption within 48 h was also significantly decreased (4.71 ± 2.71 mg vs 7.25 ± 3.76 mg, *p* = 0.006). The application of RSB reduced opioid consumption by nearly 35%. From the view of opioid consumption, we can conclude that combined PIFB with RSB could provide better analgesia, although pain scores were similar (at rest *p* = 0.287, 0.653, 0.449, at cough *p* = 0.097, 0.551,0.371) at 12, 24, 48 h between the two groups. As all participants were educated by professional staff on how to use analgesic devices according to their own pain intensity and demands. There was no significant difference in the incidence of pain at the chest tube within 48 h (14.8% vs 29.6%, *p* = 0.190), only a higher trend in the PIFB group. There was no difference in the adverse events with opioids, perhaps hydromorphone is a kind of improved opioid subtype with fewer adverse events.

Cardiac surgery is commonly performed via median sternotomy, which causes catastrophic pain, particularly sternal splitting. Full heparinization and hemodynamic instability make the use of epidural analgesia or paravertebral block controversial. Transverse thoracic muscle plane block and PIFB, aiming at the anterior chest wall innervated by branches of intercostal nerves, can achieve the same analgesia for sternotomy in cardiac surgery [9], but PIFB is more superficial, safer and simpler. [10] While pain after cardiac surgery is complicated, drainage insertion can cause skin incisions to rub. In addition, irritation of drainage to adjacent tissues and rectus abdominal muscle would result in persistent pain that cannot be covered by PIFB [8], so additional methods should be combined for better pain management. The most painful area related to chest tubes was mainly concentrated in the epigastric area [11]. RSB is widely used in laparoscopic surgery and targets upper abdominal postoperative analgesia [12]. We conducted a randomized controlled trial to prove that RSB combined with PIFB is a more optimized maneuver to provide adequate analgesia compared to single PIFB in cardiac sternotomy.

The duration of a single shot for regional anesthesia is limited, even for long-acting local anesthetics. Surprisingly, a single shot of PIFB combined with RSB in our trial decreased opioid consumption until 48 h after surgery. The long duration of combined regional techniques can be explained as follows. The addition of dexamethasone, as a safe and effective adjunct, can further prolong the duration of long-acting local anesthetics [13]. Its effect on sensory block duration is dose-independent between 4 and 10 mg [14]. In our study, patients in the PIFB + RSB group received 10 mg dexamethasone, which contributes to sustained analgesia. 

There was no difference in time to drainage removal, ability to ambulate, or LOS in the ICU and hospital between the two groups. Although opioid consumption was decreased in our trial, the effect on early recovery was mild. Actually, the process of recovery is related to a series of programs, including adequate postoperative analgesia, surgeon-based preferences, and protocols for system perioperative care [15]. The implementation of enhanced recovery after surgery requires the participation and cooperation of all staff and patients. Perhaps continuous blocks would show more benefits than a single shot. Continuous bilateral erector spine plane block or infusion of local anesthetics at the median sternotomy site has been identified to reduce opioid consumption and LOS in hospitals after cardiac surgery [16, 17]. Continuous PIFB combined with RSB, covering T_1_-T_10_, has been attempted with good analgesia in cardiac surgery. [18] More research is needed to find the association between postoperative analgesia and early recovery in cardiac surgery in the future.

Sawing the sternum and cardiopulmonary bypass in cardiac surgery made patients experience severe systemic inflammation associated with poor outcomes. IL-6, IL-10 and TNF-α are important cytokines that are related to surgical trauma and the degree of tissue damage, and the levels of these cytokines reflect the systemic inflammatory response to some extent [19–21]. In our study, the combination of PIFB and RSB did not attenuate perioperative systemic inflammation. There was no difference in the level of these cytokines, which may be the basis for the same clinical outcome in the two groups.

Chest tube-related pain has been recognized gradually, and this pain is described as piercing and occurring upon breathing and coughing. In addition, the pain related to chest tubes is severe and persistent, and many protocols have been administered to address this problem. Injection of bupivacaine into the pleural and mediastinal drains has been concluded to relieve pain after cardiac surgery [22]. Intrapleural injection of lidocaine can also reduce drainage pain and improve pulmonary function after CABG [23], and even topically administered lidocaine could be useful [6, 24]. The safety of these methods is uncertain due to the probability of arrhythmia or wound infection caused by local anesthetics. RSB is an ultrasound-guided direct regional technique with safety and definite analgesia. Moreover, it has been combined with PIFB to manage subxiphoid drainage and sternal pain successfully in an awake patient undergoing cardiac surgery debridement [25]. Most researches in cardiac field merely solve partial postoperative pain with a single nerve block, while we combine regional techniques for better and adequate analgesia after cardiac median sternotomy.

There are some limitations in this trial. First, PIFB and RSB were conducted after anesthesia induction to maximize patient comfort, so we cannot check the spread range of regional blocks according to the patients’ sense. Successful nerve block was uncertain merely from total intraoperative opioid consumption and hemodynamics. However, all regional blocks were guided under ultrasound, and the spread of the drug was definitely observed. Second, our sample size was based on the primary outcome, and it was small to detect the differences in postoperative early outcomes. However, we conducted a randomized controlled trial to explore the efficacy of PIFB combined with RSB. Combined regional techniques could provide adequate analgesia for median sternotomy in cardiac surgery.

## Conclusions

PIFB combined with RSB decreased intravenous opioid consumption and provided better analgesia after cardiac surgery.

## Data Availability

Original data are available from the corresponding author on request.

## Abbreviations

ASA: American Society of Anesthesiologists
CABG: Coronary artery bypass grafting
ICU: Intensive care unit
IL: Interleukin
LOS: Length of stay
NRS: Numeric rating scale
NYHA: New York Heart Association;
PCIA: Patient controlled intravenous analgesia
PONV: Postoperative nausea and vomiting
PIFB: Pecto-intercostal fascial block
RSB: Rectus sheath block
TNF: Tumor necrosis factor
TTMPB: Transverse thoracic muscle plane block
